# Numerical study of crude oil batch mixing in a long channel

**DOI:** 10.1007/s12182-018-0276-4

**Published:** 2018-12-06

**Authors:** H. Sepehr, P. Nikrityuk, D. Breakey, R. S. Sanders

**Affiliations:** Department of Chemical and Materials Engineering, Donadeo Innovation Centre for Engineering, University of Alberta, Edmonton, AB T6G 1H9 Canada

**Keywords:** Mixing, Turbulence, LES, RANS

## Abstract

The main objective of this work is to predict the mixing of two different miscible oils in a very long channel. The background to this problem relates to the mixing of heavy and light oil in a pipeline. As a first step, a 2D channel with an aspect ratio of 250 is considered. The batch-mixing of two miscible crude oils with different viscosities and densities is modeled using an unsteady laminar model and unsteady RANS model available in the commercial CFD solver ANSYS-Fluent. For a comparison, a LES model was used for a 3D version of the 2D channel. The distinguishing feature of this work is the Lagrangian coordinate system utilized to set no-slip wall boundary conditions. The global CFD model has been validated against classical analytical solutions. Excellent agreement has been achieved. Simulations were carried out for a Reynolds number of 6300 (calculated using light oil properties) and a Schmidt number of $~10^4$. The results show that, in contrast to the unsteady RANS model, the LES and unsteady laminar models produce comparable mixing dynamics for two oils in the channel. Analysis of simulations also shows that, for a channel length of 100 m and a height of 0.4 m, the complete mixing of two oils across the channel has not been achieved. We showed that the mixing zone consists of the three different mixing sub-zones, which have been identified using the averaged mass fraction of the heavy oil along the flow direction. The first sub-zone corresponds to the main front propagation area with a length of several heights of the channel. The second and third sub-zones are characterized by so-called shear-flow-driven mixing due to the Kelvin–Helmholtz vortices occurring between oils in the axial direction. It was observed that the third sub-zone has a steeper mass fraction gradient of the heavy oil in the axial direction in comparison with the second sub-zone, which corresponds to the flow-averaged mass fraction of 0.5 for the heavy oil.

## Introduction

The mixing of two-liquid miscible flows has attracted a great deal of attention due to its relevance to practical applications, e.g., mixing liquids using centerline injectors (Cao et al. 2003), improving pipe wall fouling mitigation and cleaning (Regner et al. 2007), and the batch transportation of crude oil with different properties (batching) (Ekambara and Joshi 2003). In batching, where crude oil batches with different qualities and properties are transported by the same pipeline, a blended zone is created at the interface of the oil batches. The volume of the blended zone grows with time. It is important to estimate the extent of the mixing and the size of the blended zone to predict the volume of high-quality light crude that will be contaminated with the lower-quality heavy crude during transportation. Ultimately, in this process, the operational question is where to cut the batches. To answer this question, we have to understand the different scenarios which can be observed during batching. One possible scenario is a case where both heavy oil (HO) and light oil (LO) flows are turbulent. In cases where a turbulent-turbulent configuration exists, a fairly well defined blended zone is created between batches for which, with a good accuracy, it is possible to say that if the mixed zone is cut in half, it contains 50% of each crude oil. Another possible scenario is a case where, due to the high viscosity of the heavy oil, the heavy oil flow is laminar, while the light oil flow is turbulent (laminar-turbulent configuration). Complications arise when the turbulent flow follows the laminar flow. In other words, when the light oil batch is transported after the heavy oil batch in the same pipeline, a complicated blended zone is created between the batches in which long tails of the heavy oil stretch into the light oil. In these cases, it is very difficult to estimate the phase mass fraction in the blended zone that is created. This study focuses on such cases.

Over the last few decades, many studies have been published on gravity- and pressure-driven two-fluid flows. Some works focused on the experimental and/or numerical investigation of miscible gravity-driven flows (Hallez and Magnaudet 2008; Séon et al. 2004, 2007a, b). For instance, Séon et al. (2004) studied the buoyant mixing of two fluids in tubes experimentally, evaluating both the tube’s angle of inclination and the contrast in density between the fluids. The fluids had identical viscosities. It was shown that buoyancy-driven mixing in tilted tubes differs significantly from that in the vertical tubes, which was investigated by Debacq et al. (2003). In particular, Séon et al. (2004) demonstrated that Kelvin–Helmoltz instabilities play a crutial role in flows mixing in tilted tubes. Later, Séon et al. (2007a, b) published more detailed studies on buoyancy-driven flows in nearly horizontal tubes, in which the dynamics of the mixing front (boundary separating two fluids) were predicted using a CFD-based model. The CFD simulations provided a criterion to distinguish between inertia-controlled or viscosity-controlled gravity-driven interpenetration flows in tilted tubes with low angles. Moreover, the authors established basic scaling laws determining the characteristic timescales and velocities of the mixing processes.

Hallez and Magnaudet (2008) carried out direct numerical simulations (DNS) to investigate the effect of the channel geometry on the evolution of the concentration and flow fields in the gravity-driven mixing of two miscible fluids in tilted tubes. They observed striking differences between the mixing dynamics in 2D and 3D geometries during the long-time evolution of the flow. In addition, they found three different regimes for the front velocity (depending on the tilt angle), which was in agreement with the results of experimental investigations (Hallez and Magnaudet 2008). However, it should be mentioned that the authors did not adequately take into account how the mixture viscosity depended on the local concentration of each fluid. In their momentum conservation equations, the kinematic viscosity was taken as a constant. Some other researchers have focused on the displacement of miscible fluids due to an imposed flow. Taghavi et al. (2010) experimentally studied how the flow rate affected the stability of a buoyant exchange flow between two miscible fluids of equal viscosity in a long tube. They measured the evolution of the front velocity ($V_{\mathrm{f}}$) as a function of the imposed velocity ($U_0$). It was found that at low values of inflow velocity, $U_0$, there was an exchange-flow-dominated regime characterized by Kelvin–Helmholtz-like instabilities. When $U_0$ increased, it was observed that the flow became stable and $V_{\mathrm{f}}$ increased linearly with $U_0$. However, at a large $U_0$, it was concluded that $V_{\mathrm{f}}$ is almost the same as $U_0$. In another study, using DNS, Sahu et al. (2009) investigated the effect of buoyancy on the dynamics of pressure-driven flow of two miscible fluids in inclined channels. They examined the effect of the density ratio, Froude number, and channel inclination on the flow dynamics for different Reynolds numbers and viscosity ratios. They showed that the rates of mixing and displacement of the more viscous fluid are enhanced as the density ratio and Froude number increase. Furthermore, these rates are shown to increase when the inclination angle increased and the displaced fluid is the denser one. The most recent research, which is closely related to the case of interest in our study, has been conducted by Taghavi and Frigaard (2013) and Taghavi et al. (2012). They presented a numerical framework for estimating the degree of mixing between successive miscible fluids pumped along a near-horizontal pipe. However, the aspect ratio of their geometry was 100 and, to reduce computational cost, they used a tube with a diameter of 19 mm. It should also be noted that in their study, to estimate the length of the blended zone, only turbulent-turbulent and laminar-laminar configurations were examined.

In conclusion, it should be emphasized that, in most of the studies on miscible two-fluid mixing available in the literature, low-*Re* buoyant-force-driven flows [laminar flows, $Re\le 500$ (Taghavi et al. 2012)] have been considered. There are relatively few numerical studies on buoyant-force-driven flows mixing in a channel for $Re>1000$ and different fluid viscosities. Due to the high computational cost and/or complexity of experiments, the geometry in most studies has been limited to channels with an aspect ratio (length/height) of about 100, e.g., see Alba et al. (2014). Therefore, the present work describes a numerical study on the batch-mixing of two miscible crude oils (laminar-turbulent case) with different viscosities and densities in a long (100 m) channel with a height of 0.4 m. By means, the simulation length is extended beyond what has been used previously by a factor of 5 (increasing the aspect ratio by a factor of 2.5). Furthermore, we compare predictions of three different models (laminar, URANS and LES) available in the commercial CFD software Ansys-Fluent 16.2 (ANSYS, Inc. 2016).

**Fig. 1:**
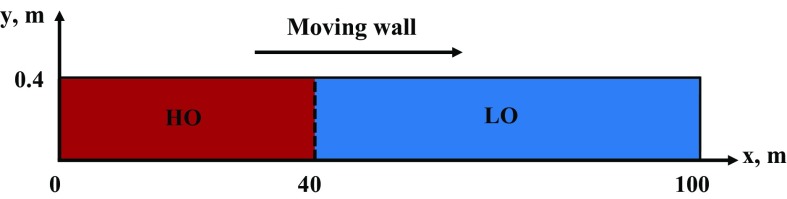
The computational domain and initial conditions

## Model formulation

Next, we describe the problem setup and CFD-based models used in our calculations. To better understand the mixing of two oils with different densities and viscosities, we consider a very long 2D channel. The length of the channel is 100 m and its height is 0.4 m, which provides an aspect ratio of 250. An illustration of the computational domain is shown in Fig. 1. The heavy oil occupies the first 40 m of the channel and light oil fills the rest (60 m). The heavy oil (HO) has a density of $924 \, {\mathrm{kg}}/{\mathrm{m}}^3$ and viscosity of 0.29 ${\mathrm{Pa\,s}}$, while the light oil (LO) has a density of 881 ${\mathrm{kg}}/{\mathrm{m}}^3$ and viscosity of 0.028 ${\mathrm{Pa\,s}}$. According to Fadaei et al. (2013), a mixture mass diffusivity of $10^{-9}$ ${\mathrm{m}}^2/{\mathrm{s}}$ is used, corresponding to the Schmidt number of $~10^4$ (calculated using the light oil properties). In this work, we consider an imposed flow with a flow-averaged velocity of 0.5 m/s corresponding to Reynolds number values of $Re=6300$ and $Re=637$, which are calculated using the light oil and heavy oil properties, respectively. Thus, it can be seen that in this case we may have a combination of turbulent and laminar flows depending on the mixing rate.

Due to the fluid properties and system conditions, four primary mixing mechanisms will dominate the two miscible fluids system:

- Mixing due to molecular diffusion (where $D_{\mathrm{m}}$ is the diffusion coefficient) since the oils are miscible.
- Mixing due to turbulent diffusion caused by the chaotic turbulent motion.
- Buoyant mixing due to the density difference between the fluids, which is induced by Rayleigh–Taylor (RT) instabilities.
- Shear-flow-driven mixing due to a velocity difference at the heavy oil–light oil interface, which is induced by Kelvin–Helmholtz (KH) instabilities.

It should be noted that though RT and KH instabilities arise from different mechanisms, they can be considered as one category. They are large-scale instabilities that introduce vorticity and enhance turbulence at the interface between the fluids. Mixing is therefore enhanced by the large-scale movement of one fluid into another as well as the fine-scale turbulence at the interface. We expect to see all four mechanisms acting in our turbulent-laminar configuration since the fluids have both density and viscosity differences.

### Unsteady laminar model

As a first approach, we use an unsteady laminar CFD-based model made up of the equation of mass and momentum conservation equations, which can be written for an incompressible flow as follows (ANSYS-Fluent, Inc. 2013):

1 $$\frac{\partial \rho }{\partial t}+\nabla \cdot (\rho \vec {u})=0$$

2 $$\frac{\partial }{\partial t} (\rho \vec {u}) + \nabla \cdot (\rho \vec {u} \otimes \vec {u}) = -\nabla p + \nabla \cdot (\mu (\nabla \vec {u}+\nabla \vec {u}^T))+ \rho \vec {g}$$

where $\rho$ is the mixture density and $\rho \vec {g}$ is the gravitational body force. In comparison with many numerical simulations, (e.g., Alba et al. 2014; Hallez and Magnaudet 2008; Taghavi and Frigaard 2013; Taghavi et al. 2010, 2012), in this work the density is not constant and depends on the local mass fraction of heavy oil. Introducing $C_{\mathrm{HO}}$ as the local mass fraction of the heavy oil (HO), the mixture density and viscosity are calculated as follows(ANSYS-Fluent, Inc. 2013):

3 $$\rho =\frac{1}{\sum _i \frac{C_{i}}{\rho _{i}}}$$

where $C_i$ is the mass fraction and $\rho _i$ is the density of species *i*.

4 $$\mu =C_{\mathrm{HO}} \cdot \mu _{\mathrm{HO}}+(1-C_{\mathrm{HO}}) \cdot \mu _{\mathrm{LO}}$$

We note that the expression (4) is well accepted in the literature approximation providing a significantly better estimate than taking $\mu$ as constant.

The conservation equation for $C_{\mathrm{HO}}$ within the unsteady laminar model has the following form:

5 $$\frac{\partial }{\partial t} (\rho C_{\mathrm{HO}}) + \nabla \cdot (\rho \vec {u} C_{\mathrm{HO}}) = \nabla \cdot (\rho D_{\mathrm{m}} \nabla C_{\mathrm{HO}})$$

6 $$C_{\mathrm{LO}} = 1 - C_{\mathrm{HO}}$$

where $D_{\mathrm{m}}$ is the mass diffusion coefficient for the heavy oil in the binary mixture. Since there are only two species in our system, we have only one mass diffusion coefficient in the equations.

It should be noted that in the commercial CFD solver ANSYS-Fluent used in this work, it is possible to select a transient solver along with the ‘laminar’ model as the viscous model. The term ‘laminar’ here implies that the Navier–Stokes equations are solved directly without any turbulence modeling. We term these selections as the ‘unsteady laminar’ model. This unsteady laminar model applied to our 2D mesh is similar to a 2D direct numerical simulation (2D DNS) in that the Navier–Stokes equations are solved along with the continuity and mass balance equations directly. However, our unsteady laminar model is different from a true 2D DNS in that it does not resolve all spatial and temporal scales. At the same time, we expect that if the mixing in the system is dominated by larger-scale motions, the result from the unsteady laminar model will be substantially the same as that obtained by 2D DNS. A grid-dependence study will be used to determine the significance of the coarser grid on the result. We note, however, that even a 2D DNS tends to underestimate mixing because it cannot capture the vortex-stretching phenomenon that contributes to the energy cascade (Ameen and Abraham 2016). In terms of the above-mentioned mechanisms, we expect that the mixing dynamic predicted by the unsteady laminar model will capture molecular diffusion and K–H and R–T instabilities, but will slightly underpredict the role of mixing due to turbulent diffusion. At this stage, we intend only to compare our selected models to the behavior expected in a pipeline-mixing scenario, to interpret the results in terms of realism and dominant mechanisms and to estimate the mixing zone size. Therefore, the unsteady laminar model is expected to be good candidate to satisfy these purposes. By choosing the unsteady laminar model, we benefit from not having to neglect or model numerical terms while at the same time reducing the computational cost for a given grid resolution.

### URANS model

The vast majority of turbulent flow computations for industrial flows are based on the RANS method due to its simplicity and low computational cost. The RANS method functions by time-averaging the Navier–Stokes equations and is capable of obtaining turbulent time-average properties (Versteeg and Malallasekera 2007). It is also possible to implement an unsteady RANS (URANS) approach that attempts to separate larger and smaller timescale motions by considering shorter time-averages of the flow properties and marching the solution through time from previous time steps. In this work, our URANS approach uses $\kappa -\varepsilon$ model as the turbulence model. Contrary to the unsteady laminar approach, in this approach the spatial and temporal turbulence scales are modeled as functions of the small-time-average mean flow properties. To accomplish this modeling, two extra equations for $\kappa$ and $\varepsilon$ are introduced to predict the Reynolds stresses and the scalar transport terms. Therefore, this approach has a significantly lower computational cost compared to the unsteady laminar approach. However, there are also several drawbacks. For instance, the underlying assumption in the $\kappa -\varepsilon$ model is that the turbulent viscosity is isotropic, which means that the ratio between Reynolds stress and mean rate of deformation is the same in all directions. This assumption fails in many complex flows. The $\kappa -\varepsilon$ model also assumes high-Reynolds-number flow that is well developed in time. We expect the effect of these assumptions on the current flow configuration to be an increase in small-scale mixing and an overestimated diffusion (molecular and turbulent) at the fluid interface. In other words, in URANS the interface will spread so that the sharp interface between phases will be less distinct. Though in principle, the KH and RT instabilities can be predicted by the URANS model, since these instabilities arise from sharp phase interfaces, we expect them to be damped significantly. The role of turbulent dispersion, however, is expected to dominate.

The $\kappa -\varepsilon$ RANS model used in this work has the following set of conservation equations (ANSYS-Fluent, Inc. 2013; Versteeg and Malallasekera 2007):

7 $$\frac{\partial \rho }{\partial t}+\nabla \cdot (\rho \vec {U})=0$$

8 $$\frac{\partial }{\partial t} (\rho \vec {U}) + \nabla \cdot (\rho \vec {U} \otimes \vec {U}) = -\nabla p + \nabla \cdot ((\mu + \mu _{\mathrm{t}})(\nabla \vec {U}+\nabla \vec {U}^T)) - \frac{2}{3}\rho \, \kappa \, {\mathbf{I}} + \rho \vec {g}$$

The turbulent viscosity is calculated as follows (ANSYS-Fluent, Inc. 2013; Versteeg and Malallasekera 2007):

9 $$\mu _{\mathrm{t}}=\rho C_{\mu } \frac{\kappa ^2}{\varepsilon }$$

10 $$\frac{\partial (\rho \kappa )}{\partial t}+\nabla \cdot (\rho \vec {U} \kappa )= \nabla \left[ \left( \mu +\frac{\mu _{\mathrm{t}}}{\sigma _{\kappa }} \nabla \kappa \right) \right] +P_k+P_b-\rho \varepsilon$$

11 $$\frac{\partial (\rho \varepsilon )}{\partial t}+\nabla \cdot (\rho \vec {U} \varepsilon )= \nabla \left[ \left( \mu +\frac{\mu _{\mathrm{t}}}{\sigma _{\varepsilon }}\right) \nabla \varepsilon \right] + C_{\epsilon 1} P_k \frac{\varepsilon }{k}(P_k+C_{\varepsilon 3}P_b)-\rho C_{\varepsilon 2} \frac{\varepsilon ^2}{k}$$

where

12 $$P_k=\mu _{\mathrm{t}} S^2, S\equiv \sqrt{2S_{ij}S_{ij}}$$

Here, *S* is the modulus of the mean rate-of-strain tensor, $P_k$ represents the generation of turbulent kinetic energy due to the mean velocity gradients and $P_b$ is responsible for the generation of turbulent kinetic energy due to buoyancy (ANSYS-Fluent, Inc. 2013). The mean strain rate $S_{ij}$ is defined as follows:

13 $$S_{ij}=\frac{1}{2}\left( \frac{\partial U_j}{\partial x_i}+\frac{\partial U_i}{\partial x_j}\right)$$

The model constants have the following values (ANSYS-Fluent, Inc. 2013): $C_{\epsilon 1} =1.44$; $C_{\epsilon 2} =1.92$; $C_\mu =0.09$; $\sigma _k=1$; $\sigma _\varepsilon =1.3$.

The conservation equation for $C_{\mathrm{HO}}$ takes the following form:

14 $$\frac{\partial }{\partial t}(\rho C_{\mathrm{HO}})+\nabla \cdot (\rho \vec {U} C_{\mathrm{HO}})= \nabla \cdot \left( \left( \rho D_{\mathrm{m}} +\frac{\mu _{\mathrm{t}}}{Sc_{\mathrm{t}}}\right) \nabla C_{\mathrm{HO}}\right)$$

where $Sc_{\mathrm{t}}$ is the turbulent Schmidt number; in this work, we used $Sc_{\mathrm{t}}=0.7$. Finally, it should be noted that the density $\rho$ and fluid viscosity $\mu$ are calculated using Eqs. (3) and (4), respectively.

### LES model

As noted already, turbulence is inherently 3D in nature, which means the turbulent flows are characterized by 3D time-dependent structures. Therefore, to examine whether the 3D mixing dynamics in our system lead to a significantly different result from that in the 2D system, we use the so-called *dynamic Smagorinsky* Large Eddy Simulation (LES) model  (ANSYS-Fluent, Inc. 2013) in a 3D version of our 2D channel. The LES approach assumes that momentum, mass and energy are transported mostly by large eddies. Therefore, only large eddies are resolved. Small eddies, however, are not resolved; instead, these small eddies and their effects on the resolved scales are modeled. Since the KH and RT instabilities are generally large scale, it is expected that their role will appear in the mixing dynamics predicted by LES. Resolving only the large eddies allows us to use coarser mesh and larger time-step sizes in LES which reduces the computational cost compared to the case with the 3D unsteady laminar model (pseudo-DNS). The dynamic Smagorinsky method differs from the standard Smagorinsky method in that the Smagorinsky constant is not set a priori but is calculated from the local dynamics of the resolved scales of motion (ANSYS, Inc. 2016).

For LES studies, we use a 3D version of our 2D channel: the length of the channel is 100 m, its height is 0.4 m, and its width is 0.024 m. Similar to the 2D case, heavy oil occupies the first 40 m of the channel and light oil fills the reminder (60 m). Basic equations of LES take the following form (ANSYS-Fluent, Inc. 2013):

15 $$\frac{\partial \rho }{\partial t}+\nabla \cdot (\rho \vec {v})=0$$

16 $$\frac{\partial }{\partial t} (\rho \vec {v}) + \nabla \cdot (\rho \vec {v}\vec {v}) = -\nabla p + \nabla \cdot \left( \left( \mu + \mu _{\mathrm{t}}\right) \left( \nabla \vec {v}+\nabla \vec {v}^T \right) \right) + \rho \vec {g}$$

17 $$\frac{\partial }{\partial t} (\rho C_{\mathrm{HO}}) + \nabla \cdot (\rho \vec {v} C_{\mathrm{HO}}) = \nabla \cdot \left( \left( \rho D_{\mathrm{m}} +\frac{\mu _{\mathrm{t}}}{Sc_{\mathrm{t}}}\right) \nabla C_{\mathrm{HO}}\right)$$

In the Smagorinsky–Lilly model, the eddy viscosity is modeled using the following relation (ANSYS-Fluent, Inc. 2013):

18 $$\mu _{\mathrm{t}}=\rho L_{\mathrm{s}}^2|\overline{S}|$$

$|\overline{S}|\equiv \sqrt{2\overline{S}_{ij} \overline{S}_{ij}}$, where $\overline{S}_{ij}$ is the rate-of-strain tensor for the resolved scale, and$L_{\mathrm{s}}$ is the mixing length for subgrid scales. For details on the LES model used in this work, we refer to ANSYS-Fluent, Inc. (2013). It should be noted that the density $\rho$ and fluid viscosity $\mu$ are calculated using Eqs. (3) and (4), respectively.

**Fig. 2:**
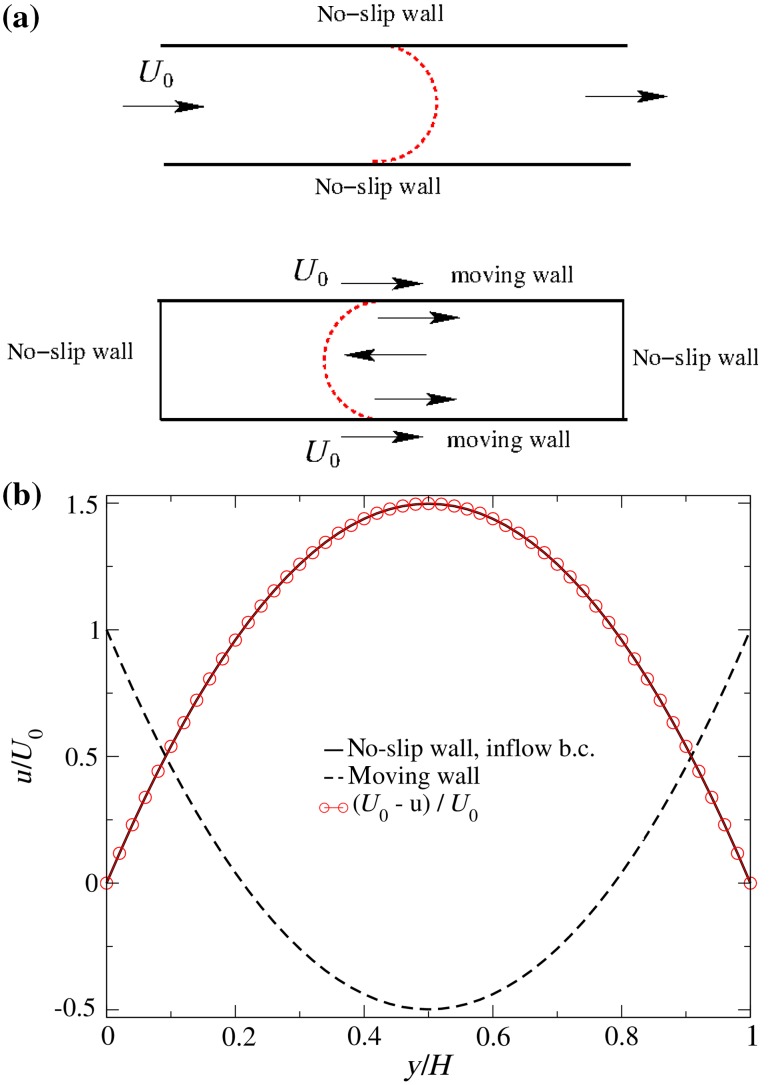
**a** Scheme of the moving-wall concept and **b** its validation against the classical approach. Here, $Re=100$ was used

**Fig. 3:**
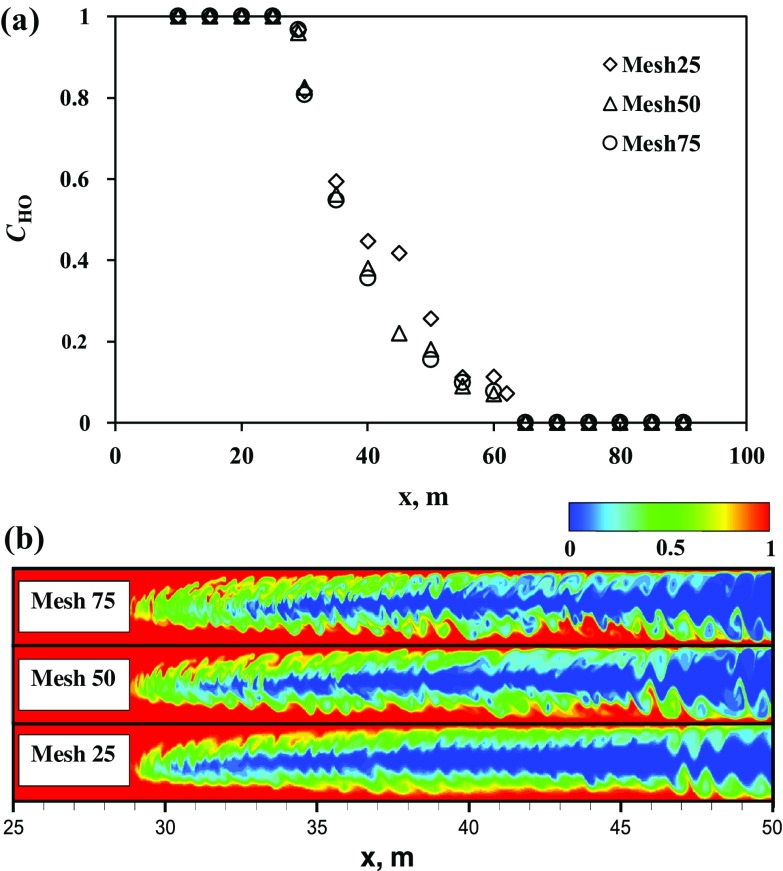
Laminar model grid studies: **a** the averaged heavy oil mass fraction along the channel calculated at $t=50$ s using different grids, see Table  1; **b** snapshots of the corresponding heavy oil mass fraction

### Boundary conditions and numerics

To model oil mixing in a 2D channel, we use the Lagrangian coordinate system, i.e., instead of having an inflow and outflow in the system, we use moving walls to generate the flow in the channel. Figure 2a shows a *moving-wall* concept and a classical approach with inlet and outlet boundary conditions. It can be seen that the moving-wall approach uses no-slip vertical end walls. The comparison of the two approaches is depicted in Fig. 2b, showing velocity profiles along the channel height. Both profiles were calculated for $Re=100$. From this figure, it can be seen that the two velocities are identical after the transformation of the moving-wall results. Finally, the following issues of the so-called moving-wall model should be emphasized:

- In contrast to a classical approach, the overall flow rate in ‘moving-wall’ model is zero
- No-slip end walls, see Fig. 3a, start to affect the instantaneous dynamics of the mixing (caused by no-slip conditions on the vertical walls) when the mixing-front approaches end walls.

In the 3D channel (LES) only the top and bottom walls move. For the side walls in the 3D channel, a symmetry boundary condition is used.

Commercial software (ANSYS, Inc. 2016) was employed to solve the problem under consideration. In detail, the governing equations for each model were solved using an implicit finite-volume technique. For pressure velocity coupling, the SIMPLE algorithm was used (Patankar 1980). The convective terms in momentum conservation and species equations for Laminar and URANS models were discretized by means of the QUICK scheme. A second-order upwind scheme was used in the $\kappa$ and $\varepsilon$ equations to discretize the convective terms. A first-order implicit scheme was activated for transient terms. For equations in the LES model, the so-called bounded central differencing scheme was used for momentum conservation equations and the MUSCL scheme for the species conservation equation. A second-order implicit scheme was chosen for the time derivative terms in the LES model. The time step in all models was set to $\Delta t=5\times10^{-3}$ s and the maximum number of iterations per time step was 40.

The Cartesian mesh used in 3D-LES simulations has dimensions of $50\times12{,}500\times3$ control volumes (H $\times$ L $\times$ W) comprising $1.9\times 10^6$ cells. For the 2D case, a grid study was carried out utilizing the three grids shown in Table 1. Results of simulations using the laminar model with the three different grids are depicted in Fig. 3a, b, which show the averaged concentration of the heavy oil $\bar{C}_{\mathrm{HO}}$ along the channel length and contour plots of ${C}_{\mathrm{HO}}$ predicted at $t=50$ s, respectively. Here, $\bar{C}_{\mathrm{HO}}$ is calculated using (Alba et al. 2014):

19 $$\bar{C}_{\mathrm{HO}}\left( t,x\right) = \frac{1}{H} \int \limits _{0}^{H} C_{\mathrm{HO}} \left( t, x,y \right) \, \hbox {d}y$$

It can be observed that for the grids ‘mesh50’ and ‘mesh75’ there were no changes in results regarding flow pattern and the averaged concentration of heavy oil. Thus, result implies that the relevant length scales to capture the overall blending dynamics are sufficiently captured by the ‘mesh50’ grid.

**Table 1:** Mesh sizes used in grid study

Name	Mesh size (H $\times$ L)	Number of CV
Mesh25	$25 \times 6250$	$0.156 \times 10^6$
Mesh50	$50 \times 12{,}500$	$0.625 \times 10^6$
Mesh75	$75 \times 18{,}750$	$1.41 \times 10^6$

## Results

Before we proceed with a description of the simulation results, let us recall the main phenomenology on buoyant miscible displacement flows in near-horizontal 2D ducts. In numerous publications, e.g., see Alba et al. (2014), Hallez and Magnaudet (2008), Taghavi et al. (2010, 2012), it was shown that after ‘an interface is opened’ separating two fluids perpendicular to the channel axis, a heavier fluid displaces a lighter fluid downwards and the front between two fluids accelerates. During the so-called *adjustment* period of time, the flow is controlled by inertia (Séon et al. 2007b) and later by viscous effects. This effect is observed in almost all displacement flows. As time progresses, Kelvin–Helmholtz (K–H) instabilities appear along the interface between the two fluids, enabling strong transverse mixing. This effect strongly depends on the density difference between the fluids, the fluid viscosities and the channel Reynolds number. It should be noted that gravity currents resulting from the release of a heavy fluid into a light fluid have similar flow scenarios. The main difference comes from the impact of the imposed flow, characterized by the Reynolds number.

According to the works  (Alba et al. 2014; Taghavi et al. 2010, 2012), the most important parameters used to classify displacement flow regimes are the Atwood number *At* and the ratio between the characteristic velocity of the displacement front (buoyancy-driven propagation of the ascending (descending) current) $V_{\mathrm{f}}$ and the imposed flow velocity $U_0$, which have the following expressions (Hallez and Magnaudet 2008):

20 $$At=\frac{\left( \rho _{\mathrm{HO}}-\rho _{\mathrm{LO}}\right) }{\left( \rho _{\mathrm{HO}}+\rho _{\mathrm{LO}}\right) }$$

21 $$V_{\mathrm{f}} \approx 0.7\sqrt{At \cdot g \cdot H}$$

Inserting the densities of the heavy oil and light oil into Eq. (20) and then Eq. (21), we obtain $At=0.024$, $V_{\mathrm{f}}=0.2$ m/s and the ratio $\frac{U_0}{V_{\mathrm{f}}}=2.5$.

**Fig. 4:**
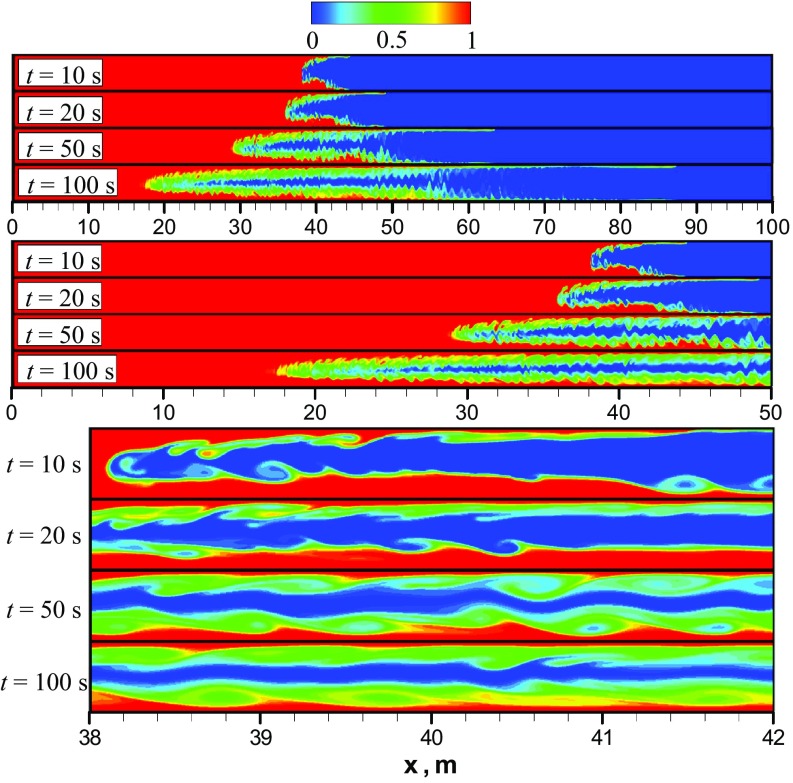
Snapshots of the heavy oil mass fraction predicted numerically using unsteady Laminar model. Here, three different zoom levels are introduced

According to the work (Taghavi et al. 2010), phenomenologically there are three different flow displacement regimes, depending on the ratio $\frac{U_0}{V_{\mathrm{f}}}$. In particular, for the parameters $At=10^{-2}$ and $\frac{U_0}{V_{\mathrm{f}}}>1$ a third regime must exist, which is characterized by negligible buoyancy forces compared to the imposed pressure gradient. For that regime, the imposed mean flow is turbulent and its interaction with the stretched interface between two fluids results in a complete displacement (Taghavi et al. 2010). Based on the current values of *At* and $\frac{U_0}{V_{\mathrm{f}}},$ we should see this third flow regime. Figure 4 shows snapshots of the heavy oil concentration predicted numerically at different times using the unsteady laminar model. To enhance the visualization of the results, we use three different zoom levels. A series of four snapshots indicates that after a short acceleration phase when the interface between two oils is enlarged in a parabolic-shape form, e.g., $t<10$ s, the K–H instabilities appear in the form of vortices which induce mixing between the two oils transversely across the channel. As a result, fingers of light and heavy oils are produced and penetrate each other in both the upper and lower layers of the flow. It should be emphasized that *the vortices resulting from K–H instability generated by the shear between heavy and light oils are not enough to mix fluids completely in the channel throughout the front propagation*, see Fig. 4 (zoomed view). We note that $C_{\mathrm{HO}}=0$ corresponds to pure light oil. As time progresses, some asymmetry can be observed in the mixing along the midplane line of the channel, as shown in the snapshots in Fig. 4. In particular, due to gravity the lower layer (comprising heavy oil) occupies a greater portion of the channel height in comparison with the upper layer. Thus, we have a structure consisting of three asymmetric layers. Similar findings were reported by Sahu et al. (2009) who used their own DNS code to simulate a two-fluid system with the density ratio of 1.5 and viscosity ratio of 2 in a 2D channel. Similar to the findings of the current study, they observed a 3-layer system with K–H instabilities. They also observed thin layers of heavy oil stretched into the lighter oil near the wall. Therefore, it can be said that, for this flow configuration, the unsteady laminar predictions conform qualitatively with expectations and that this model can capture the types of the large-scale phenomenon that occur in two-liquid miscible flows.

**Fig. 5:**
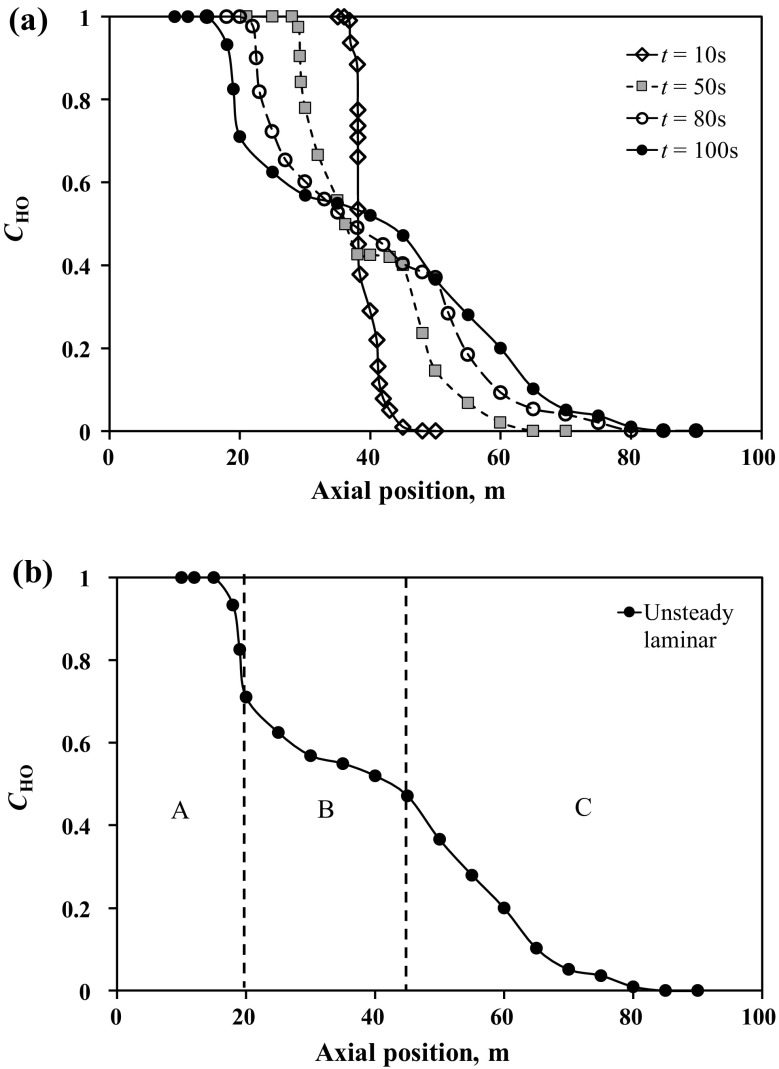
**a** Axial profiles of the height-averaged mass fraction of the heavy oil predicted numerically using laminar model at different times; **b** different mixing zones predicted using laminar model at $t=100$ s

To get a better understanding of the mixing dynamics, Fig. 5a presents axial profiles of $C_{\mathrm{HO}}$ calculated for different times using distribution of $C_{\mathrm{HO}}$ in the channel, see Fig. 4. Analysis of Fig. 5a shows that the mixing zone corresponding to $0< C_{\mathrm{HO}} < 1$ consists of the three different sub-zones, which can be more clearly observed in Fig. 5b. The first sub-zone **(A)** corresponds to the main front propagation area with a length of several heights (up to 10H) of the channel. In this sub-zone, the mass fraction in the head of the light oil results from a balance between the local mixing (molecular diffusion) and the pumping rate (convection). The second **(B)** and third **(C)** sub-zones are mainly characterized by so-called shear-flow-driven mixing due to the Kelvin–Helmholtz vortices occurring between the two oils transversely across the channel. The boundary between the first and second sub-zones is characterized by a large axial gradient of CHO. The reason for such high concentration gradient can be explained based on the fact that the mixture mass diffusivity in the system is very small ($10^{-9}$ m$^2$/s). Therefore, concentration gradient should be high to compensate for the small diffusion coefficient to balance the pumping rate. The transition between the last two sub-zones corresponds approximately to the axial coordinate when $C_{\mathrm{HO}} \le 0.5$. From Fig. 5b, it can be seen that the third sub-zone has a steeper mass fraction gradient of the heavy oil in the axial direction in comparison with the second sub-zone. The steeper gradient in the third sub-zone is due to the fact that in this sub-zone there is a very thin layer of heavy oil near the walls. Thus, K–H vortices and turbulence are suppressed in this region. The flow at these relatively late stages is expected to be dominated by diffusion. As a result, the mixing rate decreases which in turn results in a higher concentration gradient. It is this change in the character of the flow, from intensely convective to diffusive, that is primarily responsible for the change in the slope between the second and the third sub-zones.

**Fig. 6:**
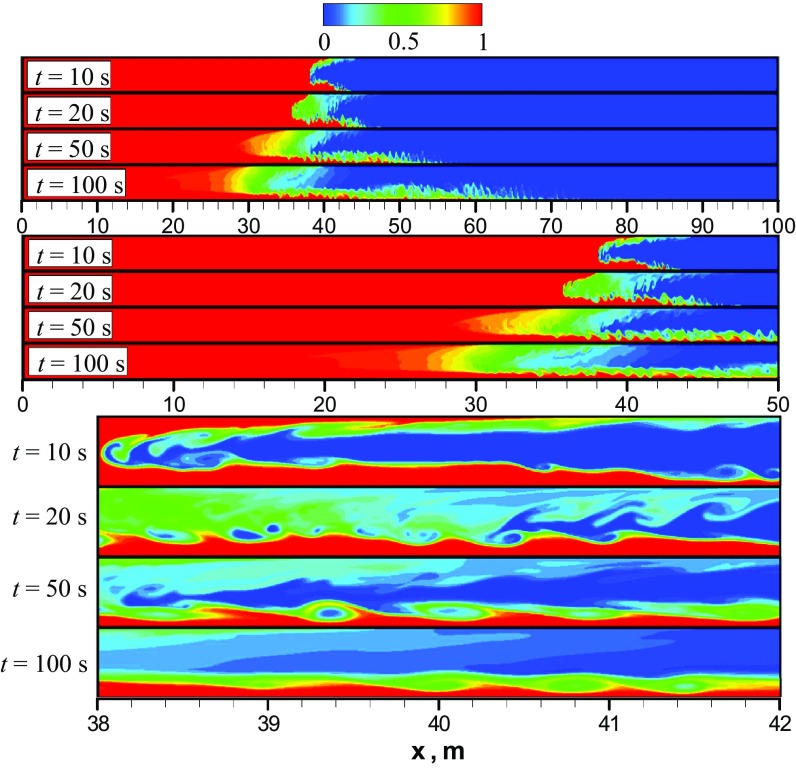
Snapshots of the heavy oil mass fraction predicted numerically using LES model. Here, three different zoom levels are introduced

**Fig. 7:**
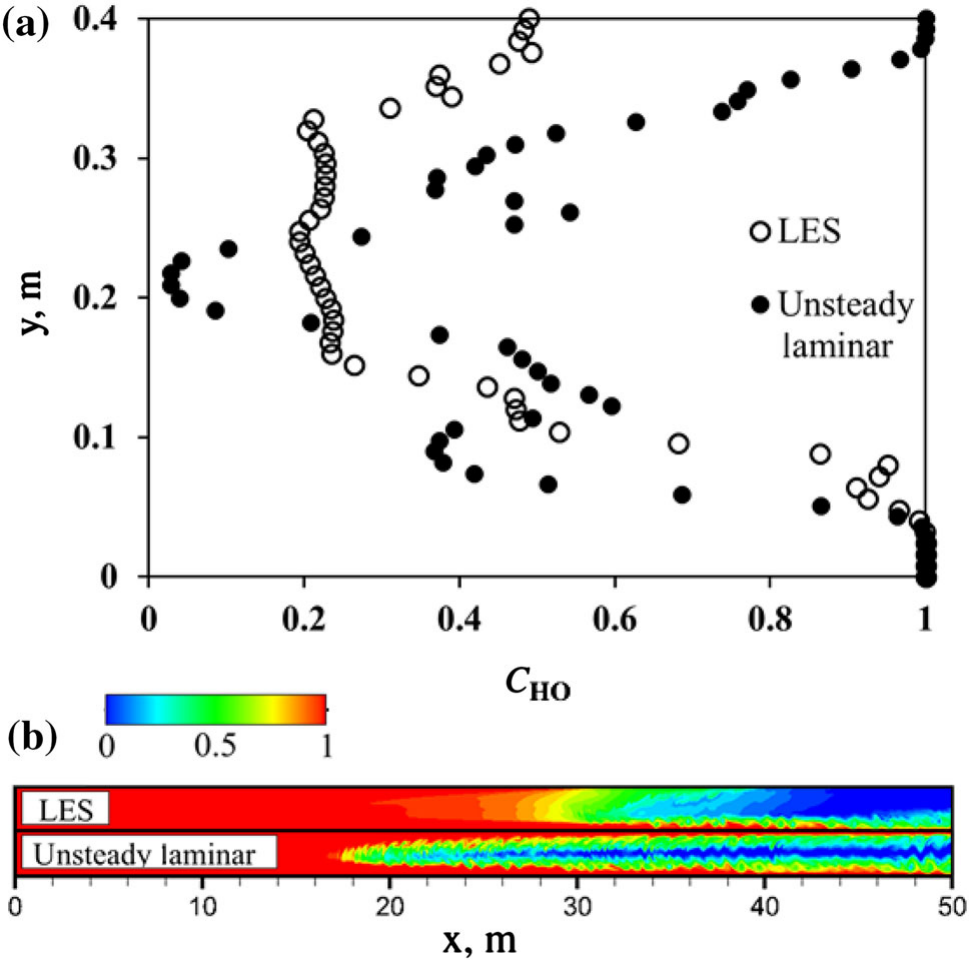
Comparison between LES and laminar models: **a** mass fraction of the heavy oil along the channel height at $x=35$ m and $t=50$ s; **b** contour plots of the heavy oil mass fraction at $t=50$ s; for clarity, only the first 50 m of the channel is shown

To illustrate the oil mixing dynamics predicted using LES, Fig. 6 depicts a series of snapshots of the heavy oil mass fraction at different times. Here, three different zoom levels for each time are introduced. A comparison of flow patterns obtained using LES and the unsteady laminar shows that during the initial adjustment time $t < 10$ s the mixing pattern is similar and mainly governed by molecular diffusion and Kelvin–Helmholtz vortices. Also, as the heavy oil in the upper part of the channel is not as thick as in the lower part, better mixing occurs in the upper part of the flow. This effect is caused by the lower viscosity of the light oil, which leads to higher turbulence in conjunction with the buoyant force pushing the turbulent fluctuations into the upper layer. The effect leads to better mixing through turbulent dispersion. In addition, we see an increased mixing effectiveness in the propagating front, and as time progresses we have a well-mixed front in comparison to the result obtained using the unsteady laminar model. Again, this increased mixing is due to increased turbulent dispersion in this region compared to the unsteady laminar simulation, which was expected since the 2D simulation damps this mixing mechanism. The effect on the concentration profile can be observed in Fig. 7, which shows profiles of CHO along the channel height calculated using LES and the unsteady laminar model at $t = 50$ s. It can be seen that in comparison with the three-layer structure predicted by the unsteady laminar model, LES gives an approximately two-layer structure of heavy oil distribution.

**Fig. 8:**
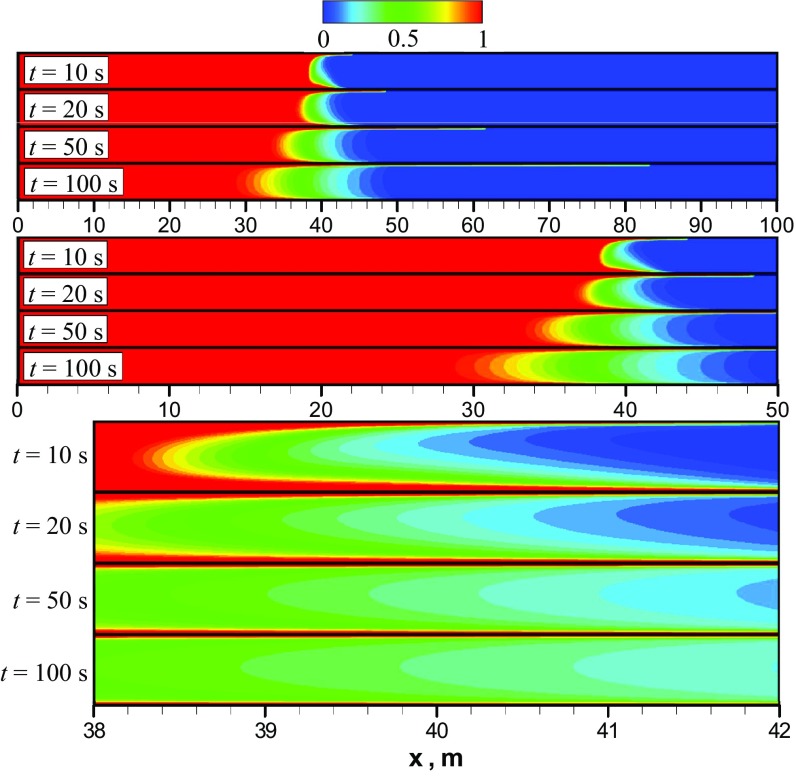
Snapshots of the heavy oil mass fraction predicted numerically using unsteady $\kappa$-$\varepsilon$ URANS model. Here, three different zoom levels are introduced

**Fig. 9:**
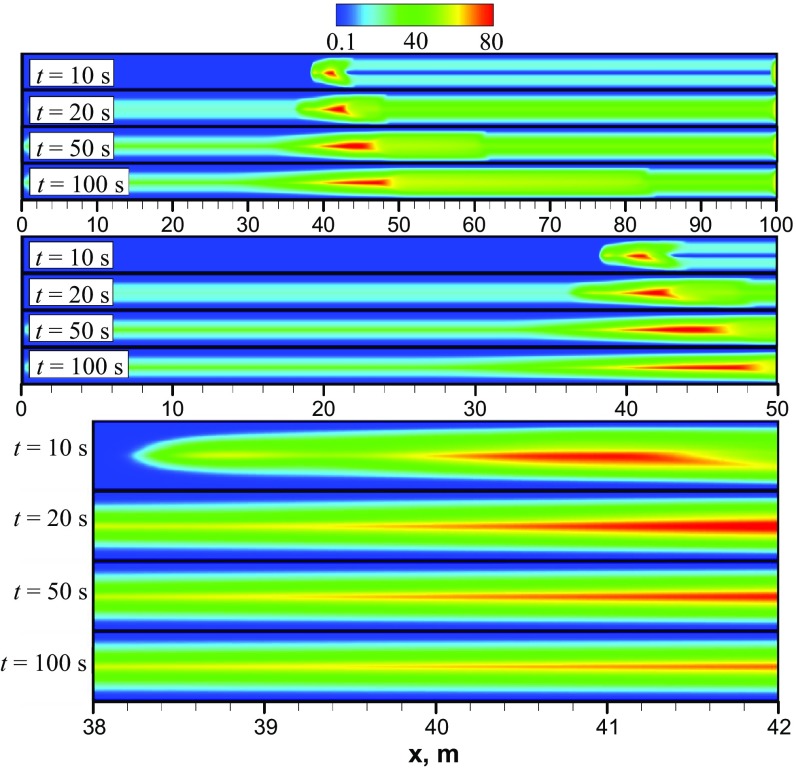
Contour plots of the turbulent viscosity ratio $\frac{\mu _{\mathrm{t}}}{\mu }$ predicted at different times using URANS. Here, three different zoom levels are introduced

**Fig. 10:**
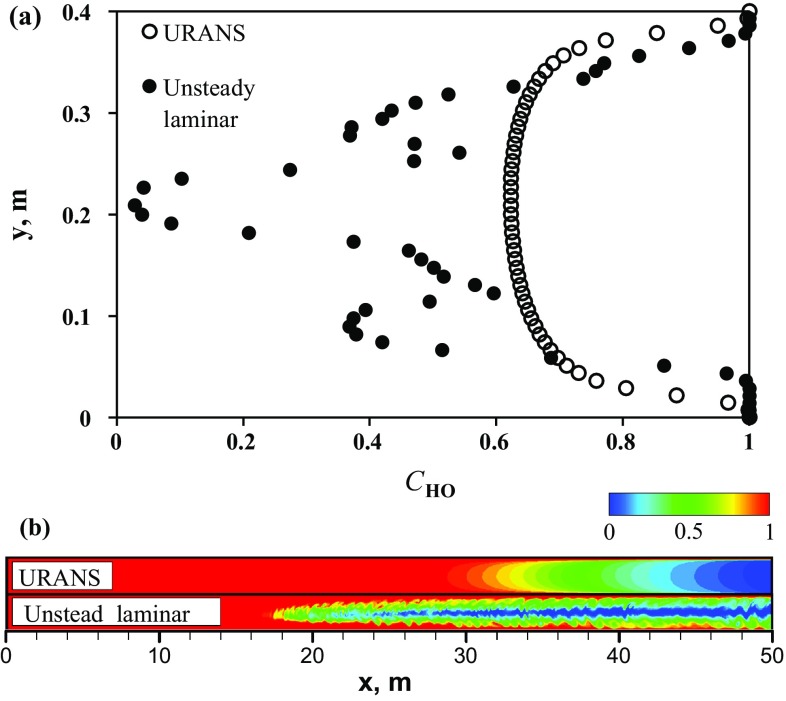
Comparison between URANS and laminar models: **a** mass fraction of the heavy oil along the channel height at $x=35$ m and $t=50$ s; **b** contour plots of the heavy oil mass fraction at $t=50$ s; for clarity, only the first 50 m of the channel is shown

**Fig. 11:**
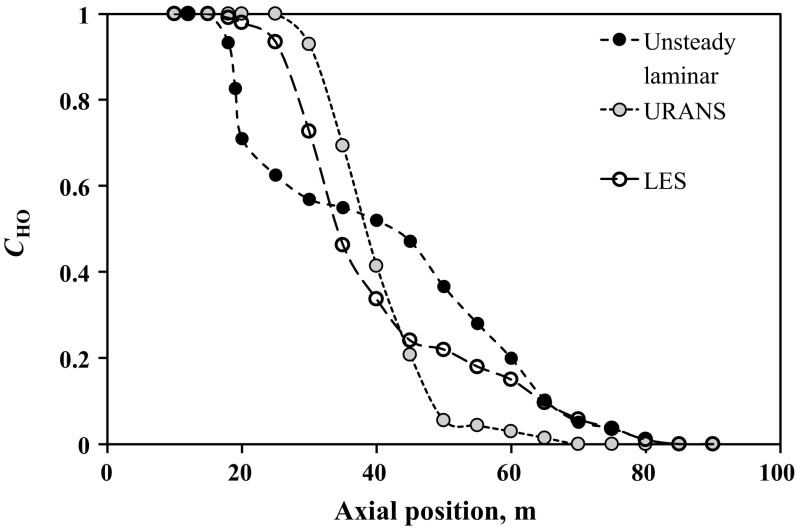
Axial profiles of the height-averaged mass fraction of the heavy oil predicted numerically using different models at $t=100$ s

Finally, Fig. 8 shows the mixing scenario obtained using the URANS model. The development of a thick mixing front can be clearly observed in series snapshots of the heavy oil mass fraction at different times. In comparison with LES and the unsteady laminar model, the mixing zone occupies nearly the entire channel height. A reasonable explanation for the mixing pattern predicted by URANS can be suggested by considering the results in Fig. 9, which shows contour plots of the turbulent viscosity ratio $\frac{\mu _{\mathrm{t}}}{\mu }$ calculated at different times using URANS. A maximum value of $\mu _{\mathrm{t}}$ is located in the mixing front, which produces a very high value for the effective diffusion coefficient $\frac{\mu _{\mathrm{t}}}{Sc_{\mathrm{t}}}>> D_{\mathrm{m}}.$ (For our system, it is 5 orders of magnitude larger.) In addition, as explained in the model description section, due to the nature of the URANS model, Rayleigh–Taylor and Kelvin–Helmholtz instabilities are not expected in concentration maps. Our simulation results from URANS confirm this. Moreover, the prediction from the URANS model conforms with the Taylor theory for the dispersion of matter in turbulent flow through a pipe. Taylor (1954) proposed a theory-based equation to estimate the mixing length between two miscible fluids with close viscosities and densities flowing in a pipe. To obtain this equation, he developed a virtual diffusion coefficient for turbulent flows, which depends on bulk flow properties and the pipe dimensions. In this virtual coefficient, the radial dispersion is 193 times greater than the longitudinal dispersion, so that vertical mixing is expected to be much more effective than axial mixing in this case. As a result, Taylor’s theory expects a fairly uniform concentration front, which is observed in these results. Moreover, this implies that the mixing length predicted by Taylor theory would be shorter than that predicted by the other models. These conclusions can be confirmed by Figs. 10 and 11. The qualitative difference between the predictions of URANS and unsteady laminar models is shown in Fig. 10. It can be seen that URANS gives slower velocities for the mixing front propagation in comparison with the unsteady laminar model. A slower and more uniform front velocity in URANS predictions is an indication of higher mixing rate in vertical direction due to turbulent dispersion than that in axial direction due to molecular diffusion.

Figure 11 compares all three models using axial profiles of the averaged mass fraction of heavy oil calculated at $t=100$ s. It can be seen that the axial profile of $\bar{C}_{\mathrm{HO}}$ predicted using URANS has a shortest mixing zone length, where $0< \bar{C}_{\mathrm{HO}}<1$, in comparison with LES and laminar models. The mixing zone length calculated using the unsteady laminar model has the maximum length, which includes three different sub-zones with different axial gradients of $\bar{C}_{\mathrm{HO}}$. The differences in the obtained mixing lengths can be understood by the discussions from the expectations for each model given in Sect. 2. We can clearly eliminate observe that URANS is unsuitable for this problem because the mixing is much more complete than we would expect for this problem, and indeed the high rate of mixing is anticipated based on the assumptions in the $\kappa -\varepsilon$ model. Differentiating between the LES and unsteady laminar simulations to determine the best option is more difficult. However, the LES appears to overestimate mixing on the side of the heavy oil when compared to the results of Sahu et al. (2009), which points to the unsteady laminar case being the most appropriate. Since this case also has a much smaller computational cost than the LES, it appears to be the best model for future work. On the side of the light oil, the LES and unsteady laminar concentration profiles are nearly identical, so it appears that the challenge is to determine the best way to resolve central finger proceeding into the heavy oil. The unsteady laminar approach appears to be the most effective to carry out this work.

## Conclusions

In this work, we used a simple no-inflow model of oil mixing with moving boundaries, to model the batch-mixing of two miscible crude oils with different viscosities and densities in a long (100 m) 2D channel. The mixing behavior of the light and heavy crude oils was investigated numerically at a Reynolds number of 6300 and Schmidt number of $10^4$. Simulations were conducted using an unsteady laminar model, URANS and LES. The results of all models showed that even a 100-m-long channel with a height of 0.4 m was not sufficient to determine a steady-state mixing zone length. The URANS model predicted the shortest mixing zone at $t=100$ s, while the laminar model showed the longest mixing zone corresponding to the channel length where $0< \bar{C}_{\mathrm{HO}} < 1$.

Using the averaged mass fraction of the heavy oil along the channel, we identified three sub-zones in the mixing zone. For the first sub-zone, in which mixing front propagation occurs, there is a very steep mass fraction gradient. In both the second and third sub-zones, there is passive mixing due to the Kelvin–Helmholtz instabilities. However, in comparison with the third sub-zone, the second sub-zone has a lower mass fraction gradient and occupies a smaller fraction of the mixing zone length, which is an indication of higher mixing in that sub-zone. Therefore, it can be said that most of the mixing occurs in the second sub-zone. The third sub-zone is the longest of the three sub-zones, which is due to the presence of long tails of the heavy oil in the light oil.

It can be concluded that the best and most efficient approach to tackle the two-fluid mixing problem is using the unsteady laminar model. Although it is a 2D simulation and contrary to a 2D DNS it filters some of the spatial and temporal scales, this approach gives us enough information about mixing dynamics in the system. The model captures the mixing mechanisms of molecular diffusion, KH and RT instabilities very well. These are the most important mechanisms observed in a laminar-turbulent configuration of the two-fluid mixing. At the same time, the unsteady laminar model takes up much less computational capacity compared to 3D LES and even 2D DNS. In addition, considering the industrial pipe flow observations explained in the introduction section, we found that the URANS is not a suitable tool to model the crude oil flow mixing as it noticeably does not capture the main mixing mechanisms affecting the mixing dynamics. On the other hand, the unsteady laminar model predictions conform with what is observed in batching.

However, we should point out that using the moving-wall approach, even with a 100-m-long channel, was not enough to determine the mixing length. Also, at higher *Re*, the spatial and temporal scales become smaller. Therefore, if we want to study the mixing dynamics at higher *Re*, finer mesh sizes will be needed to capture these scales. To extend this study, we can conduct simulations in longer 2D channels with finer grid size using the unsteady laminar model. In future work, it will be necessary to conduct the simulations in a 3D pipe geometry to fully quantify 3D mixing effects. For that work, LES will be required because it is able to capture the important mixing mechanisms and its computational cost is significantly less than 3D unsteady laminar or 3D DNS.

## List of symbols

*C*: Mass fraction
*D*: Diffusion coefficient
*H*: Channel height
$$\vec {g}$$: Gravitational acceleration
*L*: Channel length
*Re*: Reynolds number
*Sc*: Schmidt number
*At*: Atwood number
*t*: Time
$\vec {u}$: Velocity vector in unsteady laminar model
$\vec {v}$: Velocity vector in LES model
$\vec {U}$: Velocity vector in URANS model
$$V_{\mathrm{f}}$$: Front velocity
$$U_0$$: Inlet velocity
$S_{ij}$: Mean rate-of-strain tensor

## Greek symbols

$\kappa$: Turbulent kinetic energy
$\epsilon$: Turbulent dissipation rate
$$\mu$$: Molecular viscosity
$$\mu _{\mathrm{t}}$$: Turbulent viscosity
$$\nu$$: Kinematic viscosity
$$\rho$$: Density

## Subscripts

t: Turbulent
HO: Heavy oil
LO: Light oil

## References

[CR1] Alba K, Taghavi SM, Frigaard IA (2014). Miscible heavy-light displacement flows in an inclined two-dimensional channel: a numerical approach. Phys. Fluids.

[CR2] Ameen MM, Abraham J (2016). Are ‘2D DNS’ results of turbulent fuel/air mixing layers useful for assessing subgrid-scale models?. Numer. Heat Transf. Part A.

[CR3] ANSYS-Fluent. Inc. ANSYS-FLUENT Theory Guide, Release 14.0; 2013

[CR4] ANSYS, Inc. ANSYS-FLUENT™ V 16.2—commercially available CFD software package based on the Finite Volume method. Southpointe, 275 Technology Drive, Canonsburg, PA 15317, USA, www.ansys.com; 2016.

[CR5] Cao Q, Ventresca AL, Sreenivas KR, Prasad AK (2003). Instability due to viscosity stratification downstream of a centerline injector. Can. J. Chem. Eng..

[CR6] Debacq M, Fanguet V, Hulin JP, Salin D, Perrin B, Hinch EJ (2003). Buoyant mixing of miscible fluids of varying viscosities in vertical tubes. Phys. Fluids.

[CR7] Ekambara K, Joshi JB (2003). Axial mixing in pipe flows: turbulent and transition regions. Chem. Eng. Sci..

[CR8] Fadaei H, Shaw JM, Sinton D (2013). Bitumen-toluene mutual diffusion coefficients using microfluidics. Energy Fuels.

[CR9] Hallez Y, Magnaudet J (2008). Effects of channel geometry on buoyancy-driven mixing. Phys. Fluids.

[CR10] Patankar S (1980). Numerical Heat Transfer Fluid Flow.

[CR11] Regner M, Henningsson M, Wiklund J, Östergren K, Trägårdh Ch (2007). Predicting the displacement of yoghurt by water in a pipe using CFD. Chem. Eng. Technol..

[CR12] Sahu K, Ding H, Valluri P, Matar O (2009). Pressure-driven miscible two-fluid channel flow with density gradients. Phys. Fluids.

[CR13] Séon T, Hulin JP, Salin D (2004). Buoyant mixing of miscible fluids in tilted tubes. Phys. Fluids.

[CR14] Séon T, Znaien J, Perrin B, Hinch EJ, Salin D, Hulin JP (2007). Front dynamics and macroscopic diffusion in buoyant mixing in a tilted tube. Phys. Fluids.

[CR15] Séon T, Znaien J, Salin D, Hulin JP, Hinch EJ, Perrin B (2007). Transient buoyancy-driven front dynamics in nearly horizontal tubes. Phys. Fluids.

[CR16] Taghavi SM, Frigaard IA (2013). Estimation of mixing volumes in buoyant miscible displacement flows along near-horizontal pipes. Can. J. Chem. Eng..

[CR17] Taghavi SM, Seon T, Martinez DM, Frigaard IA (2010). Influence of an imposed flow on the stability of a gravity current in a near horizontal duct. Phys. Fluids.

[CR18] Taghavi SM, Alba K, Frigaard IA (2012). Buoyant miscible displacement flows at moderate viscosity ratios and low atwood numbers in near-horizontal ducts. Chem. Eng. Sci..

[CR19] Taylor G (1954). The dispersion of matter in turbulent flow through a pipe. Proc. R. Soc. Lond. Ser. A. Math. Phys. Sci..

[CR20] Versteeg HK, Malallasekera W (2007). An Introduction to Computational Fluid Dynamics. The Finite Volumem Method.

